# Pregnancy Induced Hypertension and Uric Acid Levels among Pregnant Women Attending Ruhengeri Referral Hospital, in Rwanda

**DOI:** 10.24248/eahrj.v5i1.650

**Published:** 2021-06-11

**Authors:** Francois Niyongabo Niyonzima, Ally Dusabimana, Jean Berchmas Mutijima

**Affiliations:** a Department of Biomedical Laboratory Sciences (BLS), Faculty of Applied Fundamental Sciences (AFS), INES - Institute of Applied Sciences, Rwanda

## Abstract

**Background:**

Pregnancy Induced Hypertension (PIH) is a common burden during pregnancy usually associoted with adverse maternal and paternal outcomes. The uric acid serum level was identified as an important biochemical marker which can predict preeclampsia, a type of PIH. This study was conducted to evaluate the effects of serum uric acid levels in association with blood pressure among pregnant women attending Ruhengeri Referral Hospital.

**Methodology:**

A cross-sectional study was designed and 80 pregnant women in different gestation trimesters participated in the study. Data was collected from September to October 2018. Digital sphygmomanometer was used to test blood pressures for participants. Blood samples were collected in red top tubes and centrifuged to obtain serum for uric acid levels. Using Humastar 80, uric acid levels were measured for each participant. Data was analysed using Statistical Package for Social Sciences (SPSS) version 23.0. Bivariate correlation was used to analyse the relationship between uric acid levels and participants’ blood pressure.

**Results:**

The median age was 27 in interquartile 23-31 ranging from 19 to 39 years. 58.75% of participants were in their 1^st^ trimester, followed by 21.25% in the 2^nd^ trimester and 20% in the 3^rd^ trimester. The prevalence of hypertension was 11.3% (7.5% for stage 1 and 3.8% for stage 2). Hyperuricemia was found in 15% of the participants. The mean of uric acid level was 7.12 ±1.86 mg/dl in the hypertensive group and 4.49 ±1.22 mg/dl in the non-hypertensive group. The study revealed a strong positive correlation between uric acid levels and systolic/diastolic blood pressure.

**Conclusion:**

High prevalence of hypertension among pregnant women was revealed. The association of hypertension and hyperuricemia was recorded with a strong correlation between blood pressures and serum uric acid levels. Examination of Uric acid levels among pregnant women should be routinely performed for early identification and management of hypertension.

## BACKGROUND

Pregnancy-Induced Hypertension (PIH) usually leads to maternal and neonatal morbidity. Developed and countries under development continue to experience PIH.^1^ Around 10% among pregnant women show PIH worldwide, while 14% was observed in Sub-Sahara African countries.^2^ A meta-analysis by Gemechu et al.^3^ highlighted a significant prevalence of PIH in Sub-Saharan Africa when compared to other continents. Pregnant women in Russia (1.7%), Kurdish (3.1%) and Somali (3.8%) had lower prevalence of PIH, compared to women in Finland (5.4%).^4^ The PIH was observed to be the most common cause of maternal mortality in Ethiopia due to poor awareness.^1^

Hypertension in pregnancy is expressed as a systolic blood pressure of 140 mmHg or greater and a diastolic blood pressure of 90 mmHg or greater. The various types of this disorder are defined according to the pre-set criteria. The preeclampsia or eclampsia is confirmed when hypertension appears in otherwise normotensive female after 20 weeks of gestation and is usually accompanied with proteinuria. Chronic hypertension is considered when hypertension appears prior to pregnancy or before the 20^th^ week of gestation.^2,5^ The pre-eclampsia superimposed on chronic hypertension is when de novo proteinuria appears after the 20^th^ week of gestation.^5^ Hyperuricemia is a strong risk factor for several maternal and perinatal complications in patients with hypertensive disorders during pregnancy.^6^ In Uganda, maternal near miss and death as outcomes of hypertension has been observed with severe preeclampsia associated with a high morbidity rate of 7.0%.^7^

Hypertension disorders among pregnant women in Rwanda take the second position among the causes of morbidity with 28.6% severity, according to Rulisaet al.^8^ Another research on maternal cases puts eclampsia as the leading cause of death at 30.7%.^9^ Sengoma et al.^10^ revealed that 2.7% of pregnant women acquire pre-eclampsia while only 1% develop eclampsia.

Additionally, hypertension in Musanze district, at Proominubus clinic has been associated with hyperuricemia (5.3%) and pre-eclampsia (2.1%).^11^ Other factors contributing to hypertension were estimated at 11.78%. The highest number of pregnant women with hypertension was observed at Gisenyi district hospital with a rate of 65%.^12^

During early pregnancy, uric acid levels fall often to 3 mg/dl or below, this is usually due to the uricosuric effects from oestrogen and from the increase in renal blood flow. Uric acid levels increase during the 3^rd^ trimester, reaching levels of up to 4-5 mg/dl by term. It is known that subjects destined to develop preeclampsia show slightly higher uric acid levels during the 1^st^ trimester in association with a relative reduction in urinary urate excretion.^13^ Each year, over 500,000 women die due to pregnancy outcomes with 99% residing in the developing world (low- and middle-income countries). Among those deaths, 10 to 15% are related to preeclampsia and eclampsia, these are types of hypertension among pregnant women.^14^ Hypertension generally account for 10% of the pregnant women with 2–8% facing complication due to the pre-eclampsia.^15^ Mboudoet al.^16^ showed that among 8.2% of hypertension case during pregnancy, 77.88% was due to pre-eclampsia, 15.38% due to gestational hypertension and 5.76% due to pre-eclampsia superimposed, only 0.96% had chronic hypertension.

Evidences from the study conducted by Essiben et al.^17^ demonstrated that patients with eclampsia had a statistically significant association with hyperuricemia with 36.7% of the pregnant women having severe preeclampsia. Another study showed that the rate of morbidity associated with pre-eclampsia is high at 7.0%.^7^ Few related studies were carried out and only one was conducted in Rwanda. The Rwandan study showed that hypertension with uric acid abnormality was prevalent among 5.3% of pregnant women.^11^ Therefore, this study was conducted to evaluate the uric acid levels in association with blood pressure among pregnant women attending Ruhengeri Referral Hospital.

## METHODS

### Ruhengeri Referral Hospital

Ruhengeri Referral Hospital is located in Musanze District of North Province in Rwanda. It was built during colonialism in 1939 as a public clinic. It served the public as a District Hospital for several years. Nowadays, this hospital officially serves as the National Referral Hospital and receives patients’ references from its neighbouring hospitals and health facilities in northwest Rwanda since 2013. In addition to 406,557 people who live in Musanze District, Ruhengeri Referral Hospital serves a population estimated to 386,078 people from surrounding districts.

The hospital is open all hours (24/7) and serves patients of all categories; Out Patients Department, Internal Medicine, Surgery, Gynaecology and Obstetrics, Accident and Emergency, Paediatrics, Anaesthesiology and Critical Care Medicine, Mental Health, Pathology Services and other Allied Services.

### Study Design

The study was a cross-sectional analysis of data collected from pregnant women at Ruhengeri Referral Hospital.

The study population consisted of voluntary participants who attended the Hospital during the study period precisely from September to October 2018. Demographics namely; age, blood pressure, and uric acid levels were recorded for all participants.

### Population and Sample Size Estimation

The population of this study was defined by participants’ pregnancy and their attendance at Ruhengeri Referral Hospital. Simple random estimation was used to choose the sample size for this study.

### Inclusion Criteria

Pregnant women who attended Ruhengeri Referral Hospital for the period of the study were requested to participate in the study. Pregnant women who voluntarily consented to participate were included in the study. For any reason in their judgments, recruited participants were free to withdraw from the study at any time.

### Exclusion Criteria

Hospital clients other than being women and pregnant were excluded from the study. Pregnant women who refused the voluntary participation were not included. Not accepting measurements designed for this study; either blood pressure or serum uric acid or/and both led to exclusion from the study.

### Ethical Consideration

Approvals were granted by both Ruhengeri Institute of Higher Education (INES Ruhengeri) and Ruhengeri Referral Hospital Ethics committees. Data for this study was collected anonymously. Confidentiality was maintained at all levels of the study. Participants were informed that their participation in the study was voluntary and that they were free to withdraw from the study at any time for any reason. The consenting during recruitment was made verbally.

### Demographics and Gestation Trimesters Data

Demographics data was collected for all recruited participants. Oral interviews were conducted to collect demographics, like age. Only females were interviewed in this study. Data for gestation trimester was collected from the participant's medical file. It was counted in weeks of gestation from week one (1) to the end of the pregnancy, usually week forty (40). It was recorded as trimester 1, 2 or 3. The first, second, and third trimester consisted of participants with pregnancy period ranging between week one (1) to week twelve (12), week thirteen (13) to week twenty-six (26), and week twenty-seven (27) to week forty (40) respectively.

### Blood Pressure Testing

Blood Pressure (BP) measurements were collected from each study participant. A digital sphygmomanometer with adults cuff sizes was used in blood pressure testing. The participants were asked to seat in a chair with their feet flat on the floor and relax for 5 minutes. The arm was rested on the table at the level of the heart. The blood pressure cuff was wrapped around the top of the arm with the bottom of the cuff above the elbow. The device was switched on and automatically provided the result in approximately one minute. Results were recorded to the data collection form in mmHg.

### Uric Acid Levels Quantification

Serum uric acid levels were quantified in the present study. Venous blood samples were collected in 4ml red top tubes with clot activators for all participants by following routine venepuncture standard procedures. Collected samples were transported to the clinical chemistry laboratory unit. Bench top laboratory centrifuge was used to separate blood serum from cells. Blood serum was quantified for uric acid levels by using a semi-automated chemistry analyser Humastar 80, strictly following the test operating procedure and instruments operation manual. Results for uric acid levels were read in mg/dL, and printed from the instrument-controlling computer. Results were validated and recorded to the data collection form.

### Data Analysis

Collected data was cleaned and analysed using the Statistical Package for Social Sciences (SPSS) version 23.0. Figures and tables were used in presenting the analysed data. Demographic data available in this study was analysed and presented. Descriptive analysis was performed for blood pressures and uric acid levels. Results for both variables were compared with reference ranges. Hypertension and hyperuricemia was categorised for blood pressures and uric acid levels, respectively. Bivariate statistics was used to determine the relationship between hypertension and elevated levels of uric acid among participants. The association of variables was considered to be statistically significant at 95% confidence interval (CI) with the *P<.05.*

## RESULTS

### Demographics and Gestation Trimesters

A total of 80 participants were recruited and included in the study (Table 1). Demographically, all participants were female with varying ages. 4 classes were made, precisely; [19–23[, [23–27[, [27–31[, and [31–40[ years, and were represented by 17.50, 26.25, 27.50, and 28.75%, respectively. The median was 27 and interquartile was 23–31. The age varied in a range of 20 years from 19 to 39 years old. From week 1 to the 12^th^ week of gestation, this period is considered the first trimester, 47 (58.75%) participants were recorded in this trimester. Gestation week 13 to week 26 is the second trimester, 17 (21.25%) participants were recorded. The third trimester was represented by 16 (20.00%) participants with weeks of gestation 27 to 40 or above. The peak number of participants was recorded in the first trimester. The 2^nd^ and the 3^rd^ trimesters of gestation almost had equally distributed participants.

**TABLE 1:** Clinical Characteristics of Participants

Characteristics	Count	Percentage
**Pregnancy trimester**		
1	47	58.75
2	17	21.25
3	16	20.0
**Mean ± SD**		
Uric acid (mg/dL)	4.78±1.54	
Diastole (mmHg)	76±6	
Systole (mmHg)	116±11	

### Blood Pressure among Study Participants

72.5% of participants in this study had normal BP results. Hypertension was found in 11.3% of participants.

(7.5% stage 1, 3.8% stage 2). No participant was found with low blood pressure. However, elevated blood pressure was observed in 16.2% of the study participants.

### Uric Acid Levels among Study Participants

According to trimesters of gestation, uric acid levels noted were 4.84, 5.03, and 4.36 for trimester 1, 2, and 3 respectively. The prevalence of 15% was observed in participants with hyperuricemia. One third of the participants were above 30 years. In the age groups of (23-27) and (27-31) years, the prevalence of hyperuricemia was a bit low at 14.3 and 13.6%, respectively. In addition, also among the women aged below 23, the rate of hyperuricemia was a bit low.

### Comparison of Serum Uric Acid Levels among Participants with or without Hypertension

In Table 2, the participating women with hypertension stage 1 and stage 2 were introduced in the hypertensive group. Normotensive and participants with only high blood pressure which had not yet developed into hypertension (specifically blood pressure of 129/80 mm Hg), were introduced in the non-hypertensive group. The mean of uric acid level reading among pregnant women with hypertension and those without hypertension was significant.

**TABLE 2:** Comparison of Uric Acid Levels between Hypertensive and Non-Hypertensive Participants

Variable	Mean	SD	F	*P-value*
**Hypertension**				
Hypertensive group	7.12	1.86	16.157^*^	<.001
Non-hypertensive group	4.49	1.22		
**Systolic blood pressure**				
Hypertensive group	135.44	8.2	4.965^*^	<.001
Non-hypertensive group	113.62	9.2		
**Diastolic blood pressure**				
Hypertensive group	87.89	3.41	1.852^*^	.027
Non-hypertensive group	74.07	4.17		

* Significant (P-value less than .05)

### Association between Uric Acid Levels and the Severity of Hypertension among Study Participants

**FIGURE 1:**
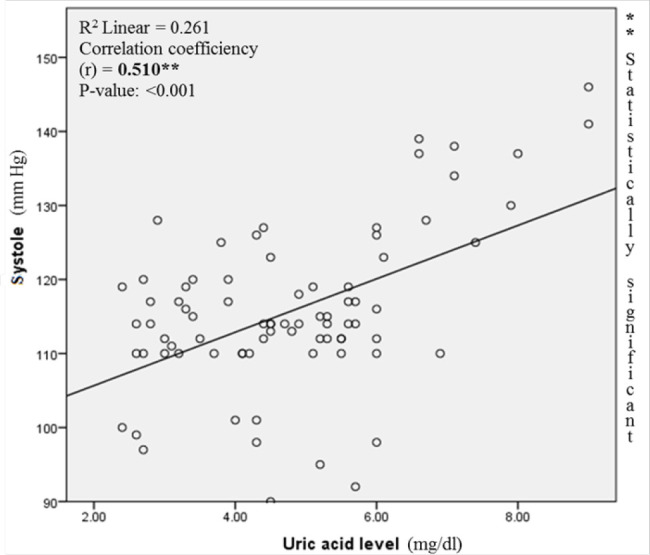
Correlation between Systolic Blood Pressure and Uric Acid Level

**FIGURE 2:**
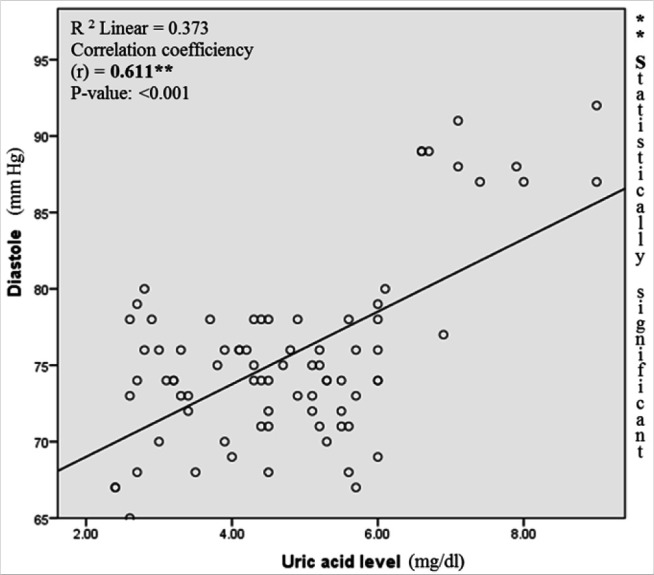
Correlation between Diastolic Blood Pressure and Uric Acid Level

The study assessed the relationship between uric acid level and the severity of hypertension. Based on a bivariate correlation and on a scatter plot, the correlation between uric acid level and systolic blood pressure (Figure 1) as well as uric acid level and diastolic blood pressure (Figure 2) were determined. A strong correlation was observed between blood pressures and serum uric acid levels.

## DISCUSSION

The uric acid levels among pregnant women with hypertension were evaluated in this study. Increase in uric acid level was observed in the 2^nd^ trimester. The mean of uric acid levels noticed in general participants was equivalent to the mean observed among the non-Pregnancy Induced Hypertension (PIH) group in a study carried out in New-Delhi.^18^ On the other hand, like observed in this study, several studies have also showed that pregnant women who attend antenatal care arrive in the 1^st^ and the last trimester, although in this study, the number was low in the third trimester.^11–19,20^

Blood Pressure is very useful in screening and diagnosing hypertension.^21^ The total prevalence rate of hypertension was 11.3% among pregnant women in this study. Leodgard found in his study a moderate prevalence rate.^11^ The prevalence observed for hypertension stage 1 (blood pressure ranging from 130 to 139 mm Hg and 80 to 89 mm Hg systolic and diastolic, respectively) and stage 2 (systolic blood pressure of 140 mm Hg or higher and diastolic blood pressure of 90 mm Hg or higher) in this study was low compared to the findings by Adu-Bonsaffohet al.^22^ whose study recorded 21.4% rate of PIH among pregnant women in Ghana and Muti et al.^23^ who found a 19.4% rate of PIH in Zimbabwe. This study found a high rate of hypertension in comparison to many studies which were reviewed. ^1,23,24,25,26^

Depending on the uricosuric of oestrogen and due to the increased glomerular filtration rate, uric acid levels fall among more than 25% of pregnant women.^27^

In this study, 15% prevalence of hyperuricemia was recorded among participants. In contrast to the findings of this study, hyperuricemia was found to be high (5.3%) in a study conducted in the same study clinic located in the same area in Rwanda^11^ and in another study conducted by Ali et al.^28^ in Bangladesh. The prevalence observed among Nigerian pregnant women was also more than twice the findings of the current study.^29^ In concordance to the study by Fawzy et al.,^27^ approximately the same prevalence among Ethiopian pregnant women was recorded. This highlight the need for assessing the demographic factors contributing to differences discussed above.

Due to its impact in predicting preeclampsia, uric acid level examination is a useful biochemical test most often performed during pregnancy.^30^ Serum uric acid levels were compared among pregnant women with and those without hypertension. The increased mean of uric acid in the hypertensive participants is in concordance to the findings observed by Khurshid et al.^31^ who observed it to be 7.29±1.24 in the preeclamptic group compared to 4.39±0.72 mg/dl in the normal pregnancy group in a study conducted in Pakistan. The study conducted in India observed 7.63±0.59 vs 4.94±0.83 mg/dl in PIH and non-PIH women respectively.^18^ Additionally, the observed significant difference between the 2 groups was also observed among pregnant women in a study conducted in Nepal.^14^ Besides, in contrast to the findings of this study, although there was also an increase of uric acid among Japanese pregnant women attending Antena tal at the National Kyushu medical centre, the observed mean uric acid level among PIH group (3.5±0.9 mg/dl) - was low. Not only uric acid in the PIH group was considered to be low, also among non-PIH group, their uric acid level was low 2.9±0.8 mg/dl.^19^ This suggests that there could be a significant effect of demographic area towards uric acid levels.

The association between uric acid levels and the severity of hypertension among pregnant women was studied and was observed. The findings of this study agree with the correlation of uric acid and SBP as well as DBP observed in Chinese healthy groups.^32^ This may confirm the high prevalence of hyperuricemia related hypertension observed by Lédgard^11^ who conducted the study in pregnant women in the Northern Province of Rwanda. This highlights the need for routine examination/analysis of uric acid levels among pregnant women to assess the burden of hypertension which is very high.

## CONCLUSION

The current study evaluated the association between hypertension and hyperuricemia among pregnant women. Stage 1 and 2 hypertensions were found to be high among participants. Hyperuricemia was also found to be high. The association of hypertension and hyperuricemia with astrong correlation was observed between blood pressures and serum uric acid levels. In addition, there was a significant difference between uric acid levels and blood pressure among the hypertensive and the non-hypertensive groups. The routine testing of uric acid levels among pregnant women, regardless their trimester of pregnancy is recommended in order to predict hypertension early.. More related studies should be conducted in all hospitals in Rwanda to generate more data that can be used by policy makers and researchers.

### Limitations of this study

The study used biochemistry standard method to measure serum uric acid levels and standard methods of blood pressure testing, thus results are valid.

-Participants in this study were from a single hospital in one district among 42 hospitals found in 30 districts in Rwanda, excluding national referrals; therefore the prevalence estimates and outcomes are not of nationwide applicability.
